# Remote Care for Caregivers of People With Psychosis: Mixed Methods Pilot Study

**DOI:** 10.2196/19497

**Published:** 2020-07-28

**Authors:** Kristin Lie Romm, Liv Nilsen, Kristine Gjermundsen, Marit Holter, Anne Fjell, Ingrid Melle, Arne Repål, Fiona Lobban

**Affiliations:** 1 Institute of Clinical Medicine, Norwegian Centre for Mental Disorders Research, Faculty of Medicine, University of Oslo Oslo Norway; 2 Division of Mental Health and Addiction Oslo University Hospital Oslo Norway; 3 Division of Mental Health and Addiction Vestfold Hospital Trust Toensberg Norway; 4 Spectrum Centre Division of Health Research Lancaster University Lancaster United Kingdom

**Keywords:** REACT, psychosis, family work, early intervention, psychoeducation, mental health service, innovation, eHealth

## Abstract

**Background:**

A reduced availability of resources has hampered the implementation of family work in psychosis. Web-based support programs have the potential to increase access to high-quality, standardized resources. This pilot study tested the Norwegian version of the Relatives Education and Coping Toolkit (REACT), a web-based United Kingdom National Health Service program in combination with phone-based support by trained family therapists.

**Objective:**

We investigated how the program was perceived by its users and identified the facilitators and barriers to its clinical implementation.

**Methods:**

Relatives of people with psychosis were offered access to REACT and to weekly family therapist support (with 1 of 2 trained family therapists) for 26 weeks. Level of distress and level of expressed emotion data were collected at baseline and after 26 weeks using the Family Questionnaire and the Relatives Stress Scale. Both family therapists and a subset of the relatives were interviewed about their experiences after completing the program.

**Results:**

During the program, relatives (n=19) had a median of 8 (range 4-11) consultations with the family therapists. Postintervention, there was a significant reduction in stress and in expressed emotions in the relatives of people with psychosis. Interviews with the relatives (n=7) and the family therapists (n=2) indicated the following themes as important—the intervention turned knowledge into action; the intervention strengthened the feeling of being involved and taken seriously by the health services; and management support and the ability for self-referral were important, while lack of reimbursement and clinician resistance to technology were barriers to implementation.

**Conclusions:**

The service was found to offer a valued clinical benefit; however, strategies that aim to engage clinicians and increase organizational support toward new technology need to be developed.

## Introduction

The relatives of people with severe mental health problems often face considerable emotional, financial, and practical problems [1,2]. Despite providing the vast majority of care for people with severe mental health problems—and thus saving society, at large, considerable costs [3]—families often receive inadequate support from the mental health care system [4,5].

Recent reviews [6-8] have concluded that family work is an effective intervention both at early and later stages of psychosis. Psychoeducational single or multiple family groups are the gold standard according to several national guidelines and best practice recommendations [9]; however, despite a generally high satisfaction with family groups, some research has shown that patients, relatives, and staff find the group format resource intensive and time consuming [10]—meeting in groups requires everyone to be at the same place at the same time for longer periods. Because the traditional multifamily group format includes the patient, because the patient may not consent to family participation, relatives are sometimes excluded.

Previous research [6] has emphasized the importance of providing support and psychoeducation to the families of individuals with psychosis; however, both the implementation of evidence-based practices and the availability of skilled psychoeducational family work staff remain limited [11]. A review [12] suggested that one reason could be that families have different needs and preferences when it comes to the timing, length, intensity, and content of the intervention. In addition, staff access to training, lack of available resources, and long distances between families and trained staff can all limit access to family support.

Web-based interventions have the potential to overcome several barriers. They are accessible across geographical areas; information, interventions, and timing can be tailored to meet the needs of the individual; and these interventions can address the needs of relatives without requiring consent from the patient. Web-based interventions have already been offered to the relatives of people with other conditions who need long-term follow-ups, including those with dementia, stroke, age-related illnesses, and brain injury [13-16]. In line with this, the protocol for the Altitudes study [17] describes a purpose-built online social networking program for caregivers of young people with psychosis. The program integrates expert and peer moderation with evidence-based psychoeducation within a single app [17]. Web-based solutions have the potential to be tailored to both needs and technology availability; however, as stated by a review [18] of web-based interventions for mental health disorders, more research is needed to conclude whether and how they ameliorate the burden of relatives.

Furthermore, the introduction of digital interventions requires change in behavior at several levels of health care services. Previous research [19,20] has shown that innovation implementation has proven to be difficult because of the multiple features of health care organizations, such as their task, workforce, leadership, and performance control and measurement systems. In-depth knowledge about how the implementation of technology is received as a method to enhance digital competencies in health care is required [21,22]. For a product or service to be engaging, it must be usable, accessible, desirable, and it must fulfill human-centered design criteria [23].

The Relatives Education and Coping Toolkit (REACT) is a guided self-management intervention for the relatives of people experiencing a recent onset of psychosis. It was developed by researchers (at the Spectrum Centre for Mental Health Research in the United Kingdom, Lancaster University, and Lancashire Care National Health Services Foundation Trust) in close collaboration with relatives and patients. The aim of this intervention was to meet the clinical recommendations from the National Institute for Health and Care Excellence to offer education and support to all relatives of individuals with psychosis [24] or bipolar disorder [25]. Early testing has shown its ability to reduce stress and increase coping strategies [26].

REACT was initially developed in paper form and is supported by members of a clinical team via telephone or email. More recently, REACT has been developed into an online toolkit through which support is offered with a moderated peer forum and direct messages. The clinical efficacy and cost effectiveness of offering this online toolkit directly to relatives is currently being tested [27], and the barriers to the implementation of REACT within a clinical service in the UK are currently being evaluated [28].

The main objective was to explore how a blended approach consisting of web-based (a Norwegian version of REACT, REACT-NOR) and phone-based support from skilled family therapists would be received when offered to the relatives of people who had recently experienced their first psychotic episode. More precisely, we wanted to accomplish the following: (1) Investigate how the service was received by relatives and the family therapists. (2) Investigate the impact of the service on relatives’ levels of distress and expressed emotions. (3) Explore the critical facilitators and potential barriers to the implementation REACT-NOR into routine clinical care.

## Methods

### Setting and Participants

This study was a mixed methods pilot study. The original REACT was translated and designed to accommodate the Norwegian setting. The study was conducted at Vestfold Hospital Trust in Norway, which has a catchment area of 240,000 people and consists of mixed urban and rural areas. The REACT-NOR web program was supported by two nurses who had also been trained in psychoeducational family therapy working in the Hospital Trust. Our initial aim was to recruit the relatives of people experiencing their first episode of psychosis. Despite considerable efforts to promote the project through written communications and oral presentations within the departments involved in the study, many clinicians did not ask relatives to participate. Because of the study’s limited timeframe, we expanded the project to include the relatives of people with a longer history of psychosis and used social media to aid recruitment.

The project was disclosed and discussed with the regional ethics committee. The committee did not regard the project as medical or health professional research as understood by law; rather, the committee saw it as an assessment of a support tool for relatives, hence the project fell outside the provisions of the Health Research Act. The local data protection officer approved the project.

### Procedure

Relatives of people experiencing psychosis were included from May 2016 until January 2017. They were either referred by the treating clinicians or self-referred after reading about the project on social media or in a newspaper. All participants were related to a person who was, at the time, in treatment for psychosis. After referral, the family therapists informed the relatives about the project and requested written informed consent. Each participant received access to REACT-NOR through a personal key consisting of a username and personal password.

### Intervention

**Figure 1:**
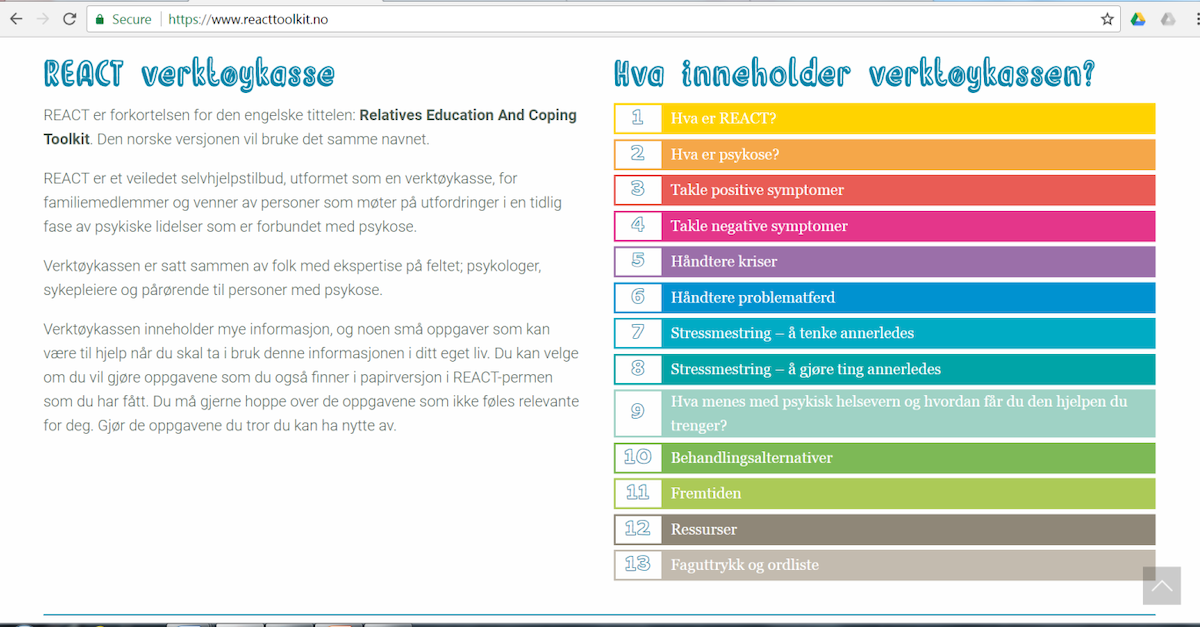
Screen capture image of the webpage with the introduction and a list of the different modules.

Since the aim of REACT is to support relatives, it does not require engagement with the person experiencing psychosis. REACT consists of 12 modules (Figure 1): (1) What is REACT? (2) What is psychosis? (3) How to handle positive symptoms; (4) How to handle negative symptoms; (5) How to handle crisis; (6) How to handle difficult behavior; (7) Coping with stress by thinking differently; (8) Coping with stress by acting differently; (9) Mental health services—How do I get the help I need? (10) Treatment options; (11) Resources; and (12) Terms and dictionary. The modules in REACT were based on psychoeducational family therapy and cognitive behavior therapy. Each module started with a psychoeducational theme, which was followed by cognitive behavior therapy–based tasks to help participants reflect on their own situation in view of what they had learned through REACT.

REACT-NOR included adjustments to reflect a Norwegian setting. The REACT dictionary was translated and customized, and the individuals in the illustrative case stories were given common Norwegian names. Relevant information about the mental health care services in Norway was added.

The program was available 24 hours a day/7 days a week, as a regular webpage. Participants could move back and forth and read relevant sections at their own pace; however, unlike the original, REACT-NOR did not provide the opportunity to interact online with the family therapists. In the original version, participants could receive support and perform the cognitive behavior therapy–based exercises online. This interactive platform could not be developed for REACT-NOR within the study’s limited budget. Instead, the participants were given a booklet containing the same cognitive behavior therapy–based exercises. The booklet was used actively during consultations with the family therapists. Support was offered by the family therapists on the phone for a maximum of 1 hour per week for 26 weeks to help participants navigate the program and to answer questions related to the program and exercises. To be proactive, the family therapists contacted the participants at least monthly if they had not responded to weekly appointment phone call or had not initiated contact themselves. Before entry into the study, each participant had a face-to-face consultation with their allocated family therapist.

### Support Staff

The 2 family therapists responsible for the support work in Norway had extensive training in psychoeducational multi- and single-family groups and used their competence when they felt it was appropriate. They were also trained to deliver support for REACT-NOR through standardized training materials provided by the research team at Spectrum Centre for Mental Health Research and through online video consultation (Skype) with trained supporters from Spectrum Centre for Mental Health Research.

### Measures

#### Quantitative Data

Expressed emotions and stress were assessed at baseline and at the end of the intervention using the Family Questionnaire [29] and the Norwegian version of the Relatives Stress Scale [30,31]. High levels of expressed emotions in relatives have been associated with higher avoidance coping, higher subjective burden, lower perceived patient interpersonal functioning, and worse outcomes [32,33]. The Family Questionnaire is a brief 20-item self-report questionnaire measuring the level of expressed emotions using a 4-point Likert-scale (never/very rarely, rarely, often, very often). It includes 10 items for criticism and 10 items for emotional overinvolvement. Higher scores represent higher levels of expressed emotions. The Relatives Stress Scale [30,31] was originally developed to measure stress in the relatives of people with dementia. The Relatives Stress Scale consists of 15 items scored on a 5-point Likert scale (never, rarely, sometimes, often, very often/always). Higher scores indicate higher levels of emotional distress, social distress, and negative feelings related to caregiving.

#### Digital Analytics

We used Google universal analytics to analyze the use of the webpage. We collected a ranking of the most visited sections in the tool based on page views (the total number of pages that had been viewed, and repeated views of one page were counted) and a list of most read sections in the tool (the mean user time spent on a particular screen).

#### Qualitative Data

After the intervention, a subset (of the relatives, n=7) was invited to take part in qualitative interviews. The 2 family therapists were also interviewed. Two couples and the therapists were interviewed as pairs; other participants were interviewed individually. All interviews were conducted face-to-face at the office of the family therapists in Vestfold Hospital Trust. These interviews were digitally recorded and transcribed by LN. The number and selection of participants and the joint group format were chosen for pragmatic purposes; there were limited resources (2- to 3-hour drive for KLR and LN between Vestfold and Oslo), and all participants had to meet during the daytime. LN carried out the interviews with KLR as a co-moderator. All interviews were conducted within 2 months of the end of the project, and each one lasted approximately 60 to 70 minutes. The interview guide was designed to capture the variety of experiences in using the REACT-NOR including general impressions, areas of specific feedback, perceived impact, and the ability to engage both relatives and family therapists. The interview guide for the family therapists included questions about their opinions on facilitators and barriers to implementation of REACT-NOR in future clinical care.

### Researchers’ Perspectives

LN is a psychiatric nurse, who, at the time of the interview, was employed by Oslo University Hospital. She has extensive clinical experience, is a trained psychoeducational family therapist, and holds a PhD in family work in first-episode psychosis. She has been collaborating with relatives and patients over many years both as a clinician and as a researcher. KLR is a psychiatrist and holds a PhD focusing on first-episode psychosis. She led the REACT-NOR pilot study and has extensive clinical experience, but no training in psychoeducational family therapy.

### Analysis

The quantitative data were analyzed with two-sided paired *t* tests. The level of significance was set to *P*=.05. When a single item was missing in one of the scales, the imputed mean for the group for that single item was used.

The qualitative data were analyzed according to the principles of systematic text condensation [34,35]. Interviews were transcribed modified verbatim (meaning that instances of “hmmm” and half sentences that were not relevant to the research question such as comments on lack of parking spaces or the temperature of the coffee were not transcribed). Analysis was conducted in 4 steps: (1) LN read through the interviews to achieve an overall impression and to look for preliminary themes related to the REACT-NOR intervention. (2) The text was broken down into manageable meaning units, and related meaning units were organized in code groups. (3) The meaning was condensed under each code group. (4) An analytic text about each category relevant for the study was developed. The transcripts were reviewed 3 or more times by LN, KLR, and KG to ensure that the data were accurately represented and interpreted. The quantitative and qualitative data were analyzed separately to distinguish contributions and to allow for different perspectives to emerge. Step 2 and step 3 were analyzed using NVivo (version 10; QSR International LLC).

## Results

### Quantitative

Using social media, we were rapidly able to recruit 19 relatives: 11 mothers, 6 fathers (including 1 stepfather), 1 sibling, and 1 spouse. Out of these, 6 were couples, and 1 was a sibling whose parents (mother and stepfather) were taking part in the project. The stepfather and the sibling withdrew during the study period because they felt it was enough to have 1 family member attending, and 1 mother found it difficult to engage in the program because her child was in the middle of a crisis and had a multitude of tasks demanding her attention; therefore, 10 mothers, 5 fathers, and 1 spouse completed the pilot. The median age was 54 (range 42-68) years, and the median duration of time spent caring for an ill relative was 12 (range 1-204) months. We lacked data on age and duration of caring time spent for 1 participant.

For 1 participant, there was no baseline data for Relatives Stress Scale; this participant was excluded from the analysis. Imputation for a missing item was applied in 3 separate cases: 1 item missing for Relatives Stress Scale at baseline, 1 for Family Questionnaire at baseline, and 1 for Relatives Stress Scale at 26 weeks.

There was a significant reduction in the level of expressed emotions from baseline to postintervention (baseline: mean 45.6, SD 7.3; postintervention: mean 42.1, SD 7.0; t_15_= 2.3, *P*=.03), with a parallel reduction in perceived level of stress (baseline: mean 24.6, SD 9.8; postintervention: mean 20.0, SD 8.6; t_14_=2.6, *P*=.02). The median number of telephone consultations with the REACT-NOR support team was 8 (range 4-11), and the telephone consultations lasted from 5-60 minutes.

The analysis of digital data found a preference for themes related to how one would handle symptoms and control stress (Table 1).

**Table 1:** User patterns from the website (highest exposure on top).

Most visited sections	Most read single pages	Mean time per page (minutes)
What is psychosis?	How to control your stress level	5.22
How to handle negative symptoms	Negative symptoms—top 10 tips	4.46
How to handle crisis	Family work	4.09
How to handle positive symptoms	The most usual thought traps	4.01
Stress—thinking differently	How can I think differently?	3.34
How to handle difficult behavior	Set up a personal plan	3.13
Stress—how to do things differently	Good advice	3.08
What do we mean by mental health care?	Guide to “the fantastic seven golden rules”	2.50
Treatment options	Usual consequences of psychosis	2.45

### Qualitative Interviews

Themes from the qualitative interviews were (1) the toolkit turns knowledge into action, (2) the service strengthened the feeling of being involved, and (3) factors important for engagement and implementation (Table 2).

**Table 2:** Themes and subthemes with exemplifying quotes.

Main themes and subthemes		Quotes
**The toolkit turns knowledge into action**		
	Educational and action oriented	“[You get]...both counseling and advice on how to best handle it. It is like asking for help in the store when you can’t find what you are looking for because you’re lost in a corner. And that’s easily done.” [mother]
	Flexibility	“You can jump back and forth [in REACT-NOR] as you need, and I really did.” [mother]
**The service strengthened the feeling of being involved**		
	Availability	“...We hope that [NN]^a^ will get well and that we won’t need any more help. But if something should happen, it is nice to know where you can look up more information so you can avoid the old traps.” [mother]
	Confidentiality	“...[as the patient refused the father to participate in the treatment]...This has been something he could accept for his own sake, so for him, it [participation without consent] was important.” [family therapist]
	Professional supporters	“...I feel it is an advantage that the supporter is a professional, yes...one with a professional background.” [mother]
	Working with personal problems	“I had to ask [family therapist] if it seemed ok how I chose to do things...you have to get some support for how you handle the situation because I didn’t know if what I was doing was normal.” [mother]
**Factors important for engagement and implementation**		
	User friendliness	“[REACT]...has a nice layout and is easy to use; everybody gave me that feedback...” [family therapist]
	Coworking on the support	“...It is important practice...and at the same time to get support from someone who knows the program.” [family therapist]
	Important to meet in person	“...When I have met her here [at the hospital], I knew that that she is real. The conversations are kind of intimate, and you don’t want to share this with anyone.” [mother]
	Management support	“People are generally positive, but it [the implementation of new interventions] is drowning in everything else that has to be remembered.” [family therapist]
	Self-referral	“I think self-referral is much better...[Ordinary referral]...will make it less available and delay the start-up.” [family therapist]
	Lacking important themes	“...[negative symptoms] could have been treated more thoroughly.” [father]

^a^For patient anonymity and privacy, this is a pseudonym.

### The Toolkit Turns Knowledge Into Action

All participants approved of the toolkit and found it easy to understand. They reported an increase in knowledge on two levels: *passive knowledge* that dealt with theoretical knowledge of the disorder, its diagnosis, and treatment; and *action-oriented knowledge* gained through completing the tasks based on their personal situation. This knowledge made them feel more in control of the situation. This was in line with the findings in Table 1. The relatives acknowledged the need for both knowledge categories, but they favored the action-oriented category which gave them practical tools:

A bit like icing on the cake, yes...it linked stress and stress management to the disease, which comes and goes. Sometimes, everything is ok, and then, there is a new breakdown, and it’s like pressing a button. The situation reactivates my own fear, but then, I can look at the worksheet again.

mother

Relatives appreciated the stepwise approach to psychoeducation, going from basic knowledge (with examples and illustrations) to in-depth knowledge; however, most relatives did not work through the toolkit systematically, but went back and forth according to their momentary needs. Service logos from Lancashire Care National Health Services Foundation Trust, Lancaster University, and Oslo University Hospital were visible in the program’s interface; this was important because it assured them that the information was trustworthy.

### The Service Strengthened the Feeling of Being Involved

All interviewed relatives had felt ignored by the mental health care system and had felt personally responsible for initiating contact and providing relevant information about symptomatology or how the person had been before the onset of illness. From their perspective, this lack of engagement acted as a barrier to an understanding of both the patient and the situation. Furthermore, this was followed by a feeling of lack of acknowledgment for the impact the disease had on their own lives:

To be involved in the treatment makes me feel that someone sees me as a person. The main focus is the person being ill, and that is how it should be, but I feel a bit ill myself sometimes because it influences my entire life...there has been quite a few limitations in what I have achieved for myself.

mother

The experiences of the relatives with REACT-NOR was in stark contrast to many of their previous experiences with health care services. They described useful and caring conversations, the feeling of being listened to, being allowed to verbalize their concerns and being able to discuss problem solving. REACT-NOR also provided them with a vocabulary that made it easier to communicate about the situation, not only with health professionals, but also with friends and family. Most relatives and the family therapists felt that a first face-to-face meeting was important; however, because 1 relative had to change family therapists during the project period, this person related that it came as a surprise that a good relationship could be achieved, even without an initial face-to-face consultation. Even though there was a median of 8 consultations, the open offer of weekly calls made relatives feel prioritized. The family therapists’ ability to offer flexible times for phone calls was valued; some participants made use of their lunch breaks or talked in the car to make time for these conversations; however, the family therapists found it challenging to make appointments. Sometimes, they had to call repeatedly, which was difficult when considering their own schedule. The relatives appreciated that participation was not dependent on consent from the patient. The option to talk freely made them feel safe. Those who would have preferred that the family therapists knew the patient argued that knowing the patient was important to be able to fully comprehend the situation and the relatives’ challenges:

...I think it is important to talk to someone who also knows the patient. Then, you know a little more about what it is all about, and you can relate to why you are in this stressful situation and why you react as you do to specific events...

mother

### Factors Important for Engagement and Implementation

#### Engagement

Both the family therapists and relatives underlined the user-friendliness of REACT-NOR and how important functioning technical solutions can be. All but the oldest participant preferred online worksheets to the booklet. Both family therapists preferred an online communication channel because this allowed them to answer questions more efficiently and to prepare for consultations; however, both family therapists and 1 relative had concerns about the self-censorship that might occur if you were to use an online messaging system:

It is even worse to write the questions online, to formulate the message, to get it right...I might extensively use the return tab; I cannot articulate it this way. It is better like this...you take a phone call and just say how it is.

mother

Several relatives had previously attended psychoeducational courses, but they had found it difficult to focus on themes that were not relevant to them at the time. A participant described their situation as being in an everlasting storm that made it difficult to remember the given information. The online format was favored because it was accessible and enabled the participants to read and reread information when needed. This was in line with the family therapists’ experience:

When working with specific chapters, one tries to support, but the relative’s concerns are often related to the sick person they are caring for and how they are coping and handling these challenges.

family therapist

All relatives pointed at the blended approach which combined family therapist support with REACT-NOR as important for their engagement. By talking to a family therapist, they received professional advice and support, and this was especially valued when they were feeling emotionally out of control and were worried if their reactions were normal or not:

There is no single element that has worked...I took a pick of what I could get, but there was one important element; this was not a friend, not close family but someone able to put things in perspective, and that was exactly what I needed.

mother

The family therapists felt that their knowledge complemented the program. Because of how the content was structured, with stepwise information and based on psychoeducation and cognitive behavior therapy, the family therapists found it easy to tailor the content to the needs of the participants. They felt that being an expert in family therapy made them more competent in meeting the relatives’ needs. One described this as follows:

I use what I know, psychoeducation, normalization, Socratic questioning, asking, reframing and identification of problems...and I believe that we have succeeded.

family therapist

Even though REACT-NOR was generally liked, there were specific themes identified in the interviews as missing and that may be relevant for engagement: (1) Having more concrete advice concerning cognitive deficits and negative symptoms because these issues were problematic for the relatives but drew little attention from the clinic; (2) a wider cultural approach to psychosis; (3) a wider range of examples of family patterns in the program than the typical nuclear family to increase representativeness and make the program more appealing to a broader group; (4) more information for siblings; (5) the family therapists missing a diagrammatic illustration of the stress/vulnerability model [36], which is widely used by family therapists in Norway; and (6) compulsory treatment being more thoroughly covered. For example:

It sounds so great you think that now he is under compulsory treatment, and then...almost everything is voluntarily anyway...[mother]

#### Implementation

The family therapists underlined the importance of management support from all levels of the organization. Introducing new ways of service delivery demands both flexibility and positive attitudes. The lack of referrals to the program was believed to be partly because of a general unfamiliarity with digital tools in mental health care. Clinicians quickly considered the relatives of the patients under their care as not suitable for the project, and some were concerned about overloading the relatives’ capacity, despite the lack of other options for structured family therapy. The family therapists suggested that this might be overcome by clear management support and the ability to self-refer. The family therapists also underlined the need for reimbursement and economic incentives as crucial to building management support. Norwegian specialist health care is reimbursed based on procedure codes, and implementation strategies must incorporate new policies for the reimbursement of remote care. Finally, the importance of having more than one family therapist at each site was considered crucial since working with new interventions makes one vulnerable because of a lack of people to share experiences with and from whom to receive support.

## Discussion

### Principal Findings

The main finding in this study was that relatives were able to receive care and be involved through a blended approach that combined a web-based intervention with support from skilled family therapists. We found some evidence of an improvement in the levels of stress (t_14_=2.6, *P*=.02) and expressed emotions (t_15_=2.3, *P*=.03). Our data confirmed that the relatives experienced REACT-NOR as a tool they could use to adjust their own behavior for both the patients and their own needs, which was in line with the results from the UK feasibility study for REACT [26]. We are not able to tease out the relative contribution of the REACT-NOR versus provision of professional support by family therapists on outcome; however, access to REACT-NOR was mentioned by both relatives and family therapists as a valuable and flexible tool that aided both information seeking and conversations. Both relatives and family therapists gave the impression that the blended approach optimized the intervention.

The ability to adjust timing and content according to their own needs was valued and was in contrast to how regular family education is offered in Norway—generally provided as a classroom teaching experience with a fixed set of themes. Family therapy is often limited because of lack of consent from the sick family member and resources. In this intervention, REACT-NOR provided relatives with both education and problem-solving skills, independent of the patient, and therefore, did not require their consent. Furthermore, the exclusive focus of the family therapists on the relatives’ needs was valued. Even though some relatives preferred that the family therapists knew the patient, the ability to feel free to receive care without concern about a breach of confidentiality was important. It may also explain the good working relationships despite the lack of regular face-to-face contact. These positive reports were similar to findings from previous research [37] which showed that relatives seemed to benefit from having the opportunity to tell their stories. Indeed, it has been reported that the relatives of people with schizophrenia are up to 10 times more likely to be socially isolated than people in the general population [38].

There were, however, areas that were not sufficiently covered. Negative symptoms and cognitive deficits required more attention; these symptoms are some of the major reasons for disability in psychotic disorders [39], and the relatives are the ones facing the problems that the symptoms cause. Treatments such as cognitive remediation, vocational rehabilitation, and cognitive behavior therapy for psychosis [40-42] could have been described in more detail in the program. A wider approach, including a multicultural understanding of psychosis and examples of mixed family patterns in the stories presented in the program may be warranted. This might be especially important because recent research underlines the different cultural norms for caregiving and in caregiving experiences [43].

It was difficult to recruit relatives through typical hospital pathways. Previous research [22] has emphasized that some of the common factors hampering the implementation of new technology is a lack of knowledge about technology. According to a meta-review by Ross et al [44], clinicians fear the loss of autonomy, have concerns about liability, and have concerns about patient privacy and security being compromised, all of which act as barriers to implementation. Clinicians may also perceive technology as a threat to the patient–health professional relationship. Ross et al [44] suggested involving the eventual users of the program in its development and implementation, improving leadership, implementing friendly and context-aware user interfaces, and providing better education. In addition, demonstrating the benefits to health professionals by having them participate in evaluating the intervention may increase their acceptance of digital interventions. We would further suggest that implementation plans for new technology in health care should include methods such as simulation training and visualization, to demonstrate how the therapy will be carried out.

The average reading time on each web page was low compared with regular services offered in a face-to-face setting; however, compared to the length of typical web-based interactions, an average time spent per page that lasts minutes, not seconds, suggests valued content [45]. More research is needed to explore how much time is needed for this type of intervention to work, highlighting the need for more knowledge about usage characteristics [46], ie, how relatives engage with the toolkit.

Furthermore, it has been suggested that the use and uptake of different types of health care services such as self-help versus professional support may be modulated by extent of the toll on mental health that they are experiencing as a result of their situation [47]. In general, it would be difficult to tell if a decline in use or a low amount of time spent using a website or web-based intervention was as a result of ineffectiveness or the opposite (ie, that the user required less help as they improved). If the intervention provided useful strategies, relatives may have been prevented from experiencing further distress, and this may have reduced their need for help. Attrition and nonuse may thus reflect the health service’s capacity to offer flexible solutions and, as such, not necessarily reflect failure. Future research should take this into account and study attrition from eHealth separately [48].

### Strengths and Limitations

The strengths of this study included the mixed method design, where we explored the perspectives of both the relatives and the clinicians; however, there were limitations. This was a small pilot study, and we were only able to include 2 family therapists. The results should be interpreted with caution regarding generalizability. The pre and postdesign with no control group did not allow us to say that the observed reduction in distress was caused by the intervention, and the sample may not have been representative, because they were chosen for pragmatic reasons. Furthermore, we were not able to recruit the relatives of people with ethnic backgrounds other than Norwegian. Change in recruitment procedure and wider inclusion criteria due to recruitment problems may have affected our results. We were not able to draw conclusions regarding relatives of patients with first-episode psychosis as we ended up with a mixed sample, and there may have been a selection bias towards female relatives who tend to be more active with regard to health information on social media such as Facebook [49].

### Conclusions

We found REACT-NOR to be an interesting method for a blended approach for therapy for families dealing with psychosis. Most families do not receive family interventions because of a lack of resources, geographical distance, or lack of consent from the patient. Web-based programs such as REACT-NOR are a valid alternative. REACT was not designed to replace other approaches, but more research should be carried out to explore how it can be used as support in blended approaches.

## Abbreviations

REACT: Relatives Education and Coping Toolkit
REACT-NOR: Norwegian version of Relatives Education and Coping Toolkit
